# Mitochondria-derived peptides in liver disease: Emerging regulators of hepatic metabolism and therapeutic targets

**DOI:** 10.1097/HC9.0000000000000885

**Published:** 2026-01-09

**Authors:** Themis Thoudam, Ge Zeng, Hui Gao, Yanchao Jiang, Nazmul Huda, Zhihong Yang, Jing Ma, Suthat Liangpunsakul

**Affiliations:** 1 Division of Gastroenterology and Hepatology, Department of Medicine, Indiana University School of Medicine, Indianapolis, Indiana, USA; 2 Department of Infectious Diseases, Nanfang Hospital, Southern Medical University, Guangzhou, Guangdong, China; 3 Department of Biochemistry and Molecular Biology, Indiana University School of Medicine, Indianapolis, Indiana, USA; 4 Roudebush Veterans Administration Medical Center, Indianapolis, Indiana, USA

**Keywords:** Humanin, liver disease, mitochondria-derived peptides, Mitochondrial Open-reading-frame of the Twelve S rRNA-c, mitochondrial DNA, SHLPs

## Abstract

Mitochondria-derived peptides (MDPs) are bioactive molecules encoded by small open reading frames within mitochondrial DNA (mtDNA). Humanin, the first MDP to be discovered, functions as a cytoprotective factor, protecting cells from stress-induced apoptosis. Subsequent discoveries expanded this family to include Mitochondrial Open-reading-frame of the Twelve S rRNA-c (MOTS-c), a key regulator of metabolic homeostasis and stress adaptation, and the Small Humanin-Like Peptides (SHLP1–6), which modulate mitochondrial bioenergetics and insulin sensitivity. MDPs play critical roles in liver homeostasis by maintaining mitochondrial function and metabolic balance. Intracellularly, they modulate mitochondrial activity, oxidative stress, and apoptosis, promoting hepatocyte survival. Extracellularly, they act in autocrine, paracrine, or endocrine manners, engaging receptors or signaling pathways to regulate nuclear gene expression and metabolic adaptation. Emerging evidence highlights their relevance in metabolic dysfunction–associated steatotic liver disease (MASLD). Humanin exerts hepatoprotective effects by inhibiting apoptosis and modulating lipid metabolism. MOTS-c activates AMPK, regulates nuclear gene expression, suppresses fibrotic and inflammatory signaling, and restores mitochondrial function in MASLD and fibrosis models. SHLPs, particularly SHLP2, enhance mitochondrial function and insulin sensitivity, supporting glucose homeostasis and mitigating oxidative stress. Collectively, MDPs establish a novel paradigm in mitochondrial signaling, extending mtDNA function beyond energy production. This review summarizes current insights into MDP biology and highlights its emerging therapeutic potential in chronic liver disease.

## Graphical abstract

**Figure:**
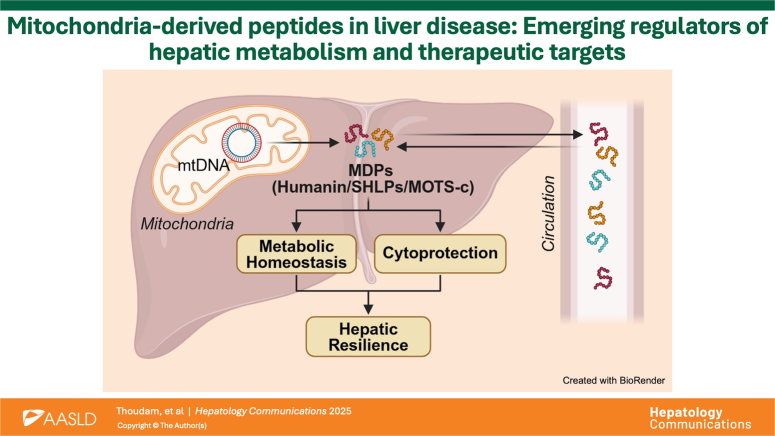

## INTRODUCTION

Mitochondria are essential organelles that serve as the primary sites of nutrient metabolism, generating the energy required to sustain cellular functions through oxidative phosphorylation (OXPHOS) and other metabolic processes.^1^ These organelles are particularly critical in metabolically active cells such as hepatocytes, where they orchestrate the metabolism of carbohydrates, fatty acids, amino acids, and xenobiotics, including alcohol, through tightly regulated biochemical pathways.^2^ The liver’s dependence on mitochondrial function underscores the central role of these organelles in maintaining hepatocellular homeostasis and overall metabolic balance.^3^ A key determinant of mitochondrial function is the integrity of mitochondrial DNA (mtDNA), which encodes essential components of the electron transport chain (ETC) necessary for efficient mitochondrial respiration.^4^

However, the role of mtDNA extends beyond energy production. Recent discoveries have identified a novel class of bioactive molecules known as mitochondria-derived peptides (MDPs), which are encoded by short open reading frames (sORFs) within mtDNA.^5^ To date, at least 8 MDPs have been described, including Humanin, Mitochondrial Open-reading-frame of the Twelve S rRNA-c (MOTS-c), and 6 Small Humanin-like Peptides (SHLP1–6).^5^ These peptides represent a new paradigm in mitochondrial biology, highlighting the broader functional significance of mtDNA in regulating cellular physiology.^6^^,^^7^ MDPs regulate a wide array of cellular processes, including mitochondrial homeostasis, oxidative stress responses, inter-organelle communication (retrograde signaling), and epigenetic regulation.^6^^,^^8^ They were initially identified through functional expression screening combined with bioinformatic analyses of mtDNA, which uncovered short coding sequences within the 16S and 12S ribosomal RNA (rRNA) regions.^9^^–^^11^ The discovery of Humanin in 2001 provided the first evidence that mitochondria produce signaling peptides with cytoprotective and metabolic regulatory functions.^9^ Subsequent identification of MOTS-c in 2015^10^ and SHLP1–6 in 2016^11^ expanded this family of mitochondrial-encoded bioactive peptides.

MDP expression closely reflects mitochondrial integrity and metabolic state. They act locally to regulate cellular signaling and mitochondrial–nuclear communication and can circulate as endocrine-like or intracrine signals influencing systemic metabolic homeostasis. These peptides exert diverse biological effects, including modulation of metabolic pathways, attenuation of oxidative and inflammatory stress, and promotion of cell survival under adverse conditions.^12^ Given their regulatory roles in cellular metabolism and stress responses, MDPs are increasingly recognized as promising therapeutic targets for liver diseases, including those driven by inflammation, steatosis, and fibrosis.^13^^–^^16^ In this review, we highlight the emerging roles of MDPs in liver health and disease and discuss their therapeutic potential in preserving hepatic homeostasis and mitigating disease progression.

## MITOCHONDRIAL DYNAMICS AND mtDNA REGULATION IN HEPATIC HOMEOSTASIS

Mitochondria are highly dynamic organelles that undergo continuous morphological remodeling through the processes of fission and fusion.^17^ These dynamic processes are critical for maintaining mitochondrial quality control, enabling adaptation to changing cellular conditions, and regulating mitochondrial number and function.^18^ Mitochondrial fission facilitates the selective elimination of damaged mitochondria via mitophagy and ensures proper distribution of healthy mitochondria during cell division.^19^^,^^20^ In contrast, mitochondrial fusion promotes the mixing of mitochondrial contents, including mtDNA, proteins, and lipids,^20^^,^^21^ which supports functional complementation and preserves mitochondrial integrity.^22^ These processes are tightly regulated and serve as essential mechanisms for maintaining cellular energy homeostasis.^23^ One of the central events coordinated during mitochondrial biogenesis is the replication and proper segregation of mtDNA into newly formed mitochondria.^24^ This ensures that each mitochondrion contains a sufficient copy number of mtDNA to maintain respiratory function.^25^ The mtDNA is a small, circular genome of ~16.6 kilobases, encoding 37 genes: 13 protein-coding genes essential for OXPHOS, 22 transfer RNAs (tRNAs), and 2 rRNAs.^26^ Unlike nuclear DNA, mtDNA lacks introns and is organized in a compact structure, with transcription initiated from the heavy strand promoter (HSP) and light strand promoter (LSP) to produce polycistronic transcripts.^26^ The transcription and maintenance of mtDNA are regulated by nuclear-encoded factors, including mitochondrial transcription factor A (TFAM) and the mitochondrial RNA polymerase (POLRMT).^27^ Translation of mtDNA-encoded proteins occurs within mitochondria on specialized mitoribosomes, utilizing mtDNA-encoded tRNAs and supported by nuclear-encoded translation machinery.^28^ The coordinated expression of mtDNA is highly responsive to intracellular cues, including metabolic status, redox balance, and inter-organelle communication.^29^ This regulation is orchestrated through nuclear–mitochondrial crosstalk, with peroxisome proliferator-activated receptor gamma coactivator 1-alpha (PGC-1α) serving as a master regulator of mitochondrial biogenesis and oxidative metabolism.^30^ Perturbations in mtDNA replication, transcription, or translation, whether due to oxidative damage, defective mitophagy, or dysregulated transcriptional control, can compromise mitochondrial function and contribute to the development of liver diseases such as metabolic dysfunction–associated steatotic liver disease (MASLD) and alcohol-associated liver disease (ALD).^3^^,^^31^^,^^32^ Given the high metabolic demand and detoxification burden placed on hepatocytes, the integrity and proper expression of mtDNA are especially critical for maintaining hepatocellular homeostasis and liver function.

## MDPs: EXPANDING THE REGULATORY ROLE OF mtDNA IN METABOLISM AND DISEASE

**FIGURE 1:**
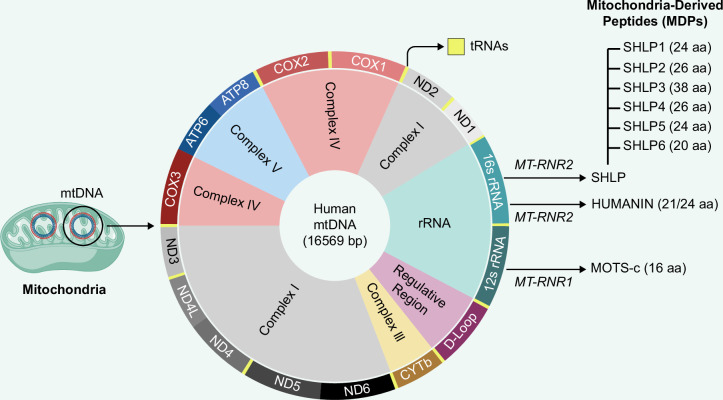
Overview of MDPs and Their Genomic Origins in mtDNA. The schematic illustrates the mitochondrial genomic loci that encode 13 ETC proteins and key MDPs, including Humanin, SHLPs, and MOTS‑c. Humanin and SHLPs are encoded by sORFs within the MT‑RNR2 region, while MOTS‑c is encoded by the MT‑RNR1 region of mtDNA. Following transcription and translation, MDPs may localize within mitochondria or be exported to the cytosol and extracellular space, acting via intracellular, autocrine, paracrine, and endocrine signaling to regulate metabolic homeostasis, stress responses, and cellular survival. Abbreviations: ETC, electron transport chain; MDPs, mitochondrial-derived peptides; MOTS-c, mitochondrial open reading frame of the 12S rRNA-c; MT-RNR1, mitochondrial 12S ribosomal RNA; MT-RNR2, mitochondrial 16S ribosomal RNA; mtDNA, mitochondrial DNA; SHLPs, small Humanin-like peptides; sORFs, short open reading frames.

In addition to its classical role in encoding essential components of the mitochondrial respiratory chain, mtDNA has recently been recognized as a source of a novel class of bioactive molecules termed MDPs, which play pivotal roles in intracellular signaling and systemic metabolic regulation.^33^ While mtDNA is traditionally known to encode 13 proteins constituting core subunits of the electron transport chain, recent studies have revealed the presence of sORFs within the mitochondrial genome.^34^ These sORFs encode previously unannotated peptides with diverse and significant regulatory functions.^35^ Among the best-characterized MDPs are Humanin, SHLPs, and MOTS-c, which are implicated in numerous physiological and pathophysiological processes.^9^^–^^11^ Humanin was initially identified through a functional expression screen of a brain cDNA library for neuroprotective activity, which mapped the peptide to the mitochondrial rRNA 2 (*MT-RNR2*, 16S rRNA) locus.^9^ This discovery subsequently led to the identification of additional MDPs, including MOTS-c, encoded within the *MT-RNR1* (12S rRNA) locus, and SHLPs within the *MT-RNR2* (16S rRNA) locus, through targeted bioinformatic searches for sORFs in mtDNA^10^^,^^11^ (Figure 1). Functionally, these MDPs regulate cellular metabolism, modulate inflammatory responses, attenuate apoptosis, and protect against oxidative and metabolic stress.^6^ Collectively, MDPs are not merely byproducts of mitochondrial transcription but active signaling molecules that expand the functional repertoire of the mitochondrial genome.

In contrast to most mitochondrial proteins, which are encoded by nuclear DNA, synthesized on cytosolic ribosomes, and imported into mitochondria via specialized translocase complexes, MDPs are encoded by sORFs within the mitochondrial genome.^36^^,^^37^ These peptides are translated either within the mitochondrial matrix or, in some cases, in the cytosol following the export of mitochondrial transcripts.^38^ Although encoded by mtDNA, many MDPs function beyond the mitochondria, being detectable in extramitochondrial compartments such as the nucleus and cytosol, as well as in the circulation.^6^ The mechanisms governing their release into circulation are not fully understood but may involve exosomes, unconventional protein secretion, or passive export during cellular stress.^39^ Once secreted, MDPs act in autocrine, paracrine, and endocrine manners by engaging cell-surface receptors, activating signaling cascades, or being internalized to influence intracellular targets.^8^^,^^40^ Through this secretory capacity, MDPs convey mitochondrial status to distant tissues and coordinate systemic responses, including metabolic adaptation, regulation of glucose and lipid homeostasis, modulation of insulin sensitivity, stress resistance, and cytoprotection against apoptosis.^7^^,^^41^

The discovery of MDPs fundamentally challenges the long-held perception of mtDNA as a static and limited genetic element whose role is confined to bioenergetics. Instead, it is now apparent that mtDNA serves as a dynamic regulatory hub, integrating metabolic cues and contributing to mitochondrial–nuclear communication as well as maintaining cellular and systemic homeostasis.^12^ MDPs represent a previously underappreciated layer of mitochondrial function, expanding the functional repertoire of the organelle beyond ATP production to include roles in adaptive stress responses, immunometabolism, and tissue protection.^7^ Understanding the regulation and function of MDPs offers promising insights into aging, metabolic diseases, and mitochondrial dysfunction-related pathologies. Elucidating the transcriptional and translational regulation of MDPs, as well as their molecular targets and signaling pathways, holds considerable promise for advancing our understanding of aging, metabolic syndromes, and mitochondrial dysfunction–associated diseases. In particular, the therapeutic potential of MDPs is gaining traction in the context of age-related disorders, insulin resistance, neurodegeneration, and chronic liver diseases such as MASLD, where mitochondrial impairment is a key pathogenic driver.^14^^,^^42^

## HUMANIN: A MULTIFUNCTIONAL MDP WITH EMERGING ROLES IN METABOLIC AND LIVER DISEASE

**FIGURE 2:**
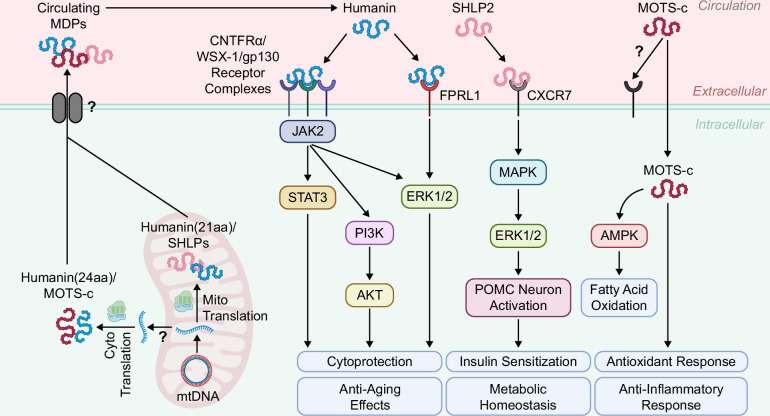
Circulating MDPs and their metabolic actions. MDPs are encoded by mtDNA. Humanin (21 amino acids, aa) is translated within mitochondria, and SHLPs are also likely translated in mitochondria, whereas Humanin (24 aa) and MOTS-c are translated in the cytosol following mRNA export from mitochondria by an unknown mechanism. MDPs are secreted into the circulation via currently undefined pathways. Circulating MDPs, Humanin, SHLP2, and MOTS-c regulate systemic metabolism. Humanin signals through CNTFRα/WSX-1/gp130 and FPRL1, activating JAK2/STAT3, PI3K/AKT, and ERK1/2 pathways, leading to cytoprotection and anti-aging effects. SHLP2 binds CXCR7, activating the MAPK/ERK1/2 pathway and hypothalamic POMC neurons, thereby enhancing insulin sensitivity and metabolic homeostasis. MOTS-c acts via cellular uptake (receptor unknown), regulating AMPK activation and fatty acid oxidation while promoting antioxidant and anti-inflammatory responses. Abbreviations: aa, amino acid; AKT, protein kinase B; AMPK, AMP-activated protein kinase; CNTFRα, ciliary neurotrophic factor receptor alpha; CXCR7, chemokine receptor type 7; ERK1/2, extracellular signal-regulated kinase 1/2; FPRL1, formyl peptide receptor-like 1; gp130, glycoprotein 130; JAK2, Janus kinase 2; MAPK, mitogen-activated protein kinase; MDPs, mitochondrial-derived peptides; MOTS-c, mitochondrial open reading frame of the 12S rRNA-c; mtDNA, mitochondrial DNA; PI3K, phosphoinositide 3-kinase; POMC, pro-opiomelanocortin; SHLPs, small Humanin-like peptides; STAT3, signal transducer and activator of transcription 3.

Humanin was the first MDP to be identified and remains one of the most well-characterized members of this emerging class of bioactive molecules.^9^^,^^43^ It is encoded by MT-RNR2 and may be translated as a 21–amino acid peptide when synthesized within mitochondria, or as a 24–amino acid peptide when translated in the cytosol, a difference attributed to the distinct genetic codes and translational machinery of the mitochondrial and cytosolic ribosomal systems^35^ (Figures 1, 2). Notably, both mitochondrial- and cytosolic-translated forms of Humanin exhibit equivalent biological activity, suggesting that its function is conserved regardless of the site of translation.^44^ In addition to its mitochondrial origin, Humanin-like peptides have been identified as products of several nuclear-encoded genes, designated MT-RNR2-like nuclear genes (MTRNR2L1–MTRNR2L13).^45^^,^^46^ These loci likely arose through the integration of nuclear mitochondrial DNA segments (NUMTs), which are fragments of mtDNA that were inserted into the nuclear genome over the course of evolution.^47^ Humanin exerts its biological effects both intracellularly and as a secreted peptide, functioning through autocrine, paracrine, and endocrine signaling pathways to protect against oxidative stress.^48^ Extracellular Humanin engages the G protein-coupled formyl peptide receptor-like 1 (FPRL1) and the ciliary neurotrophic factor receptor α/WSX-1/glycoprotein 130 (CNTFRα/WSX-1/gp130) receptor complexes, activating Janus kinase 2/signal transducer and activator of transcription 3 (JAK2/STAT3), phosphoinositide 3-kinase/protein kinase B (PI3K/AKT), and extracellular signal-regulated kinase 1/2 (ERK1/2) signaling cascades that mediate its neuroprotective effects^49^^–^^51^ (Figure 2). Intracellularly, Humanin mediates cytoprotection primarily by inhibiting apoptotic pathways.^44^^,^^52^^,^^53^ Collectively, these findings highlight a regulatory role for circulating Humanin in orchestrating receptor-mediated signaling to maintain cellular homeostasis and promote cytoprotection.

Early studies on the biological function of Humanin highlighted its potent neuroprotective properties, particularly in the context of neurodegenerative diseases such as Alzheimer’s disease (AD). In a triple-transgenic mouse model of AD—harboring the Swedish mutation in amyloid precursor protein (APPswe), a P310L mutation in tau (tauP310L), and an M146V mutation in presenilin 1 (PS-1M146V), treatment with a synthetic Humanin analogue (S14G-HN) significantly reduced amyloid-beta plaque accumulation and improved cognitive performance.^54^ These findings support the therapeutic potential of S14G-HN in targeting core pathological features of AD. Beyond its neuroprotective effects, Humanin has emerged as a key modulator of metabolic health and age-associated physiological decline. Circulating levels of Humanin have been shown to decrease with aging in both humans and animal models.^55^^,^^56^ Notably, administration of Humanin via both central and peripheral routes significantly enhances insulin sensitivity, particularly in hepatic tissue, through activation of hypothalamic STAT3 signaling.^55^ In addition, studies in *Caenorhabditis elegans* have demonstrated that Humanin treatment extends lifespan, supporting a conserved role for this MDP in promoting longevity and mitigating age-related metabolic dysfunction.^56^ Collectively, these findings underscore the critical role of Humanin, with reduced circulating levels strongly associated with the development of type 2 diabetes, AD, and other age-related disorders.^55^^–^^57^

**FIGURE 3:**
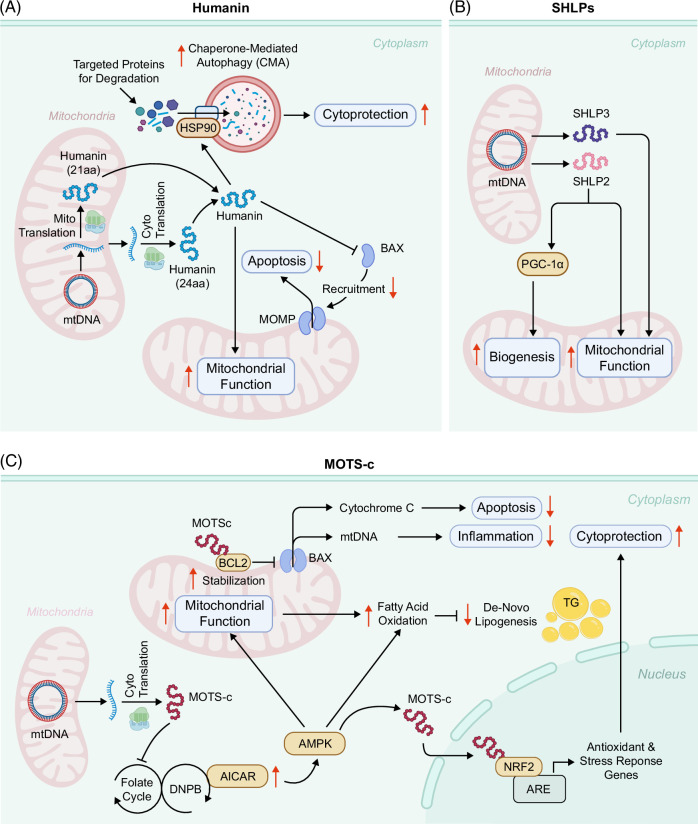
Intracellular regulatory functions of MDPs in cellular homeostasis. (A) Humanin exists in 2 forms, 24 and 21 amino acids (aa), translated from mtDNA in the cytosol and mitochondria, respectively. It inhibits apoptosis by preventing BAX recruitment to mitochondria, thereby blocking MOMP and preserving mitochondrial integrity and function. Humanin also promotes CMA via HSP90, facilitating the targeted degradation of stress-related proteins to protect cells from damage and death. (B) SHLPs, including SHLP2 and SHLP3, are encoded by mtDNA to support mitochondrial function. SHLP2, in particular, promotes mitochondrial biogenesis through activation of PGC-1α. Collectively, these peptides maintain mitochondrial health and confer cytoprotection. (C) MOTS-c, encoded by mtDNA and translated in the cytosol, enhances fatty acid oxidation and suppresses de novo lipogenesis, reducing intracellular TG levels. Specifically, it inhibits folate cycle enzymes, impairing DNPB and increasing intracellular AICAR, which activates AMPK to promote fatty acid oxidation and facilitate nuclear translocation of MOTS-c. In the nucleus, MOTS-c interacts with transcription factors such as NRF2 to regulate ARE-driven genes, thereby enhancing cellular antioxidant defense and promoting cytoprotection. In addition, it stabilizes BCL2 to block BAX-mediated MOMP, preventing cytochrome *c* and mtDNA release, thereby reducing apoptosis and inflammation. Abbreviations: AICAR, 5-aminoimidazole-4-carboxamide ribonucleotide; AMPK, AMP-activated protein kinase; ARE, antioxidant response element; BAX, BCL2-associated X protein; BCL2, B-cell lymphoma 2; CMA, chaperone-mediated autophagy; DNPB, de novo purine biosynthesis; HSP90, heat shock protein 90; MOMP, mitochondrial outer membrane permeabilization; MOTS-c, mitochondrial open reading frame of the 12S rRNA-c; mtDNA, mitochondrial DNA; NRF2, nuclear factor erythroid 2–related factor 2; PGC-1α, peroxisome proliferator-activated receptor gamma coactivator 1-alpha; SHLP2/3, small Humanin-like peptide 2/3; SHLPs, small Humanin-like peptides; TG, triglycerides.

Mechanistic studies have elucidated that Humanin confers cytoprotection, in part, through its potent anti-apoptotic actions. A critical target of Humanin is the pro-apoptotic B-cell lymphoma 2 (BCL2) family member, BCL2-associated X protein (BAX), a central mediator of mitochondrial outer membrane permeabilization (MOMP) and intrinsic apoptosis.^58^ Upon exposure to apoptotic stimuli, Humanin interacts directly with BAX, inhibiting its conformational activation and preventing its translocation to the mitochondrial outer membrane.^44^ This interference effectively blocks downstream mitochondrial events, including cytochrome *c* release and caspase activation, ultimately preserving mitochondrial integrity and enhancing cellular survival.^44^^,^^52^^,^^53^ In addition to its role in apoptosis inhibition, Humanin has been shown to protect various cell types against oxidative stress–induced injury through the activation of chaperone-mediated autophagy (CMA), a selective lysosomal degradation pathway essential for cellular quality control.^59^ In cultured cell lines such as H9C2 cardiomyoblasts, NIH3T3 fibroblasts, and MN9D dopaminergic neurons, Humanin activates CMA by interacting with heat shock protein 90 (HSP90) at the lysosomal membrane, thereby facilitating the recognition and translocation of substrate proteins for degradation.^59^ Pharmacological inhibition of HSP90 abrogates Humanin-induced CMA, confirming the dependence of this cytoprotective pathway on HSP90-mediated delivery mechanisms.^59^ These findings further substantiate Humanin’s multifaceted protective role in maintaining cellular homeostasis under stress conditions (Figure 3A).

In the context of liver disease, particularly metabolic dysfunction–associated fatty liver disease (MAFLD), Humanin has emerged as a promising MDP with significant hepatoprotective effects demonstrated in both in vitro and in vivo experimental models.^15^^,^^60^ In a murine model of diet-induced steatosis, C57BL/6J mice fed a high-fat diet (HFD) and treated with the Humanin analogue HNG (S14G-Humanin) exhibited marked improvements in hepatic and systemic metabolic parameters.^60^ Specifically, HNG administration significantly reduced hepatic triglyceride (TG) accumulation, attenuated body weight gain, and lowered visceral adiposity.^60^ These metabolic improvements were linked to enhanced activity of hepatic microsomal triglyceride transfer protein (MTTP), a key mediator of very-low-density lipoprotein (VLDL) assembly and secretion, ultimately leading to increased TG export and reduced hepatic TG accumulation.^60^ Both intravenous and intracerebroventricular HNG acutely stimulated hepatic TG secretion, a response that was abolished by vagotomy. This finding suggests that Humanin regulates hepatic lipid metabolism through a central nervous system (CNS)-mediated pathway involving the hypothalamus and vagus nerve, rather than through a neuroendocrine axis.^60^

**FIGURE 4:**
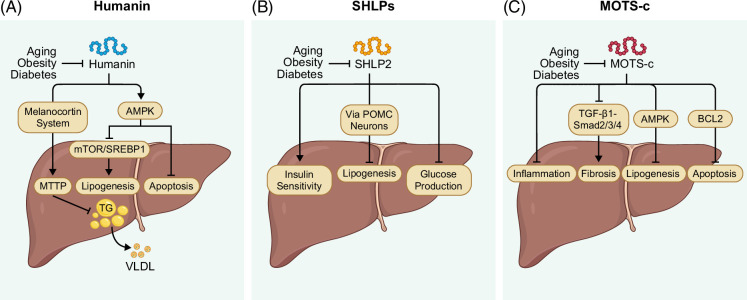
Pathophysiological roles of MDPs in metabolic and liver diseases. Downregulation of MDPs has been associated with aging, obesity, and diabetes. (A) Humanin mitigates these conditions, which are major risk factors for liver disease, by activating AMPK to reduce TG accumulation through inhibition of mTOR/SREBP1-mediated lipogenesis and by preventing apoptosis. It also downregulates MTTP and, via activation of the melanocortin system, promotes hepatic VLDL secretion, thereby decreasing intrahepatic lipid accumulation. (B) SHLP2 contributes to metabolic balance by acting on hypothalamic POMC neurons, reducing hepatic lipogenesis, enhancing insulin sensitivity, and decreasing glucose production. (C) MOTS-c exerts metabolic and hepatoprotective effects by inhibiting the TGF-β1–Smad2/3/4 pathway, reducing liver fibrosis and inflammation. It also activates AMPK to suppress lipogenesis and stabilizes BCL2 to prevent apoptosis. Abbreviations: AMPK, AMP-activated protein kinase; BCL2, B-cell lymphoma 2; MDPs, mitochondrial-derived peptides; MOTS-c, mitochondrial open reading frame of the 12S rRNA-c; mTOR, mammalian target of rapamycin; MTTP, microsomal triglyceride transfer protein; POMC, pro-opiomelanocortin; SHLP2, small Humanin-like peptide 2; Smad2/3/4, mothers against decapentaplegic homolog 2, 3, and 4; SREBP1, sterol regulatory element-binding protein 1; TGF-β1, transforming growth factor-beta 1; VLDL, very-low-density lipoprotein.

In addition, in vitro studies using primary human hepatocytes further substantiated the direct cytoprotective role of Humanin in hepatocytes exposed to lipotoxic stress. Treatment with Humanin prevented palmitate-induced intracellular lipid accumulation, apoptosis, and insulin resistance.^15^ Mechanistically, these protective effects were mediated via activation of AMP-activated protein kinase (AMPK), which in turn suppresses the mammalian target of rapamycin (mTOR)/sterol regulatory element-binding protein 1 (SREBP1) signaling axis, a pathway known to promote hepatic lipogenesis.^15^ Activation of AMPK by Humanin led to downregulation of key lipogenic genes, including SREBP1, fatty acid synthase (FAS), and stearoyl-CoA desaturase 1 (SCD1), while simultaneously enhancing insulin signaling pathways.^15^ Notably, the beneficial effects of Humanin were completely abrogated following AMPK knockdown, confirming the pivotal role of this energy-sensing kinase in mediating Humanin’s hepatoprotective actions^15^ (Figure 4A).

Collectively, these findings highlight the multifaceted regulatory functions of Humanin in hepatic lipid metabolism and insulin sensitivity, positioning it as a compelling candidate for therapeutic development in MAFLD and other liver diseases characterized by metabolic dysfunction and cellular stress, including ALD. Moreover, given its ability to act through both peripheral and CNS-mediated mechanisms, Humanin represents a novel class of endogenous mitochondrial signals with systemic metabolic implications. Future investigations aimed at delineating its receptor interactions, downstream signaling networks, and long-term safety profiles are warranted to further define its translational potential in human liver disease. These insights may ultimately facilitate the development of mitochondrial-targeted therapeutics to mitigate hepatic steatosis, improve insulin responsiveness, and preserve overall liver function in at-risk patient populations.

## SHLPs: A MDP FAMILY WITH EMERGING ROLES IN HEPATIC AND METABOLIC REGULATION

SHLPs represent a distinct family of 6 bioactive peptides (SHLP1–SHLP6), ranging from 20 to 38 amino acids in length, which are encoded by sORFs within the *MT-RNR2* locus^11^ (Figure 1). These peptides exhibit notable functional similarities with Humanin, particularly in the regulation of cell survival and metabolism, while possessing distinct amino acid sequences, tissue-specific expression patterns, and unique physiological roles.^11^^,^^61^ However, the precise site of SHLPs translation, whether in the mitochondria or the cytosol, has not been definitively investigated. The mitochondrial genomic origin of SHLPs has been firmly established through studies in HeLa-ρ0 cells, which lack mtDNA and exhibit complete loss of SHLP expression.^11^ This absence confirms that SHLPs are exclusively encoded by the mitochondrial genome and not by nuclear-encoded mitochondrial pseudogenes or NUMTs.^11^ Tissue-specific expression analyses have revealed that SHLP1, SHLP2, SHLP4, and SHLP6 are abundantly expressed in the liver, suggesting a potential physiological role in hepatic metabolic regulation.^11^ Among these, SHLP2 and SHLP3 have emerged as key regulators of systemic metabolic homeostasis.^11^^,^^62^ Importantly, both SHLP2 and SHLP3 have been shown to improve mitochondrial function, attenuate reactive oxygen species (ROS) production, and suppress apoptosis, thereby highlighting their critical roles in maintaining cellular energy homeostasis and promoting cell survival.^11^^,^^63^ Notably, SHLP2 has been shown to improve mitochondrial function in an in vitro model of age-related macular degeneration (AMD), employing ARPE-19 cybrid cells, a cellular system that replicates mitochondrial dysfunction characteristic of aging-associated diseases.^64^ In this model, SHLP2 restored mitochondrial health by normalizing mtDNA copy number and enhancing the expression of PGC-1α, a master regulator of mitochondrial biogenesis^64^ (Figure 3B). This was accompanied by increased activity of OXPHOS complexes, indicating an enhancement of mitochondrial respiration and energy production.^64^ These findings are particularly relevant in the context of MAFLD. During the progression of MAFLD to metabolic dysfunction–associated steatohepatitis (MASH), impaired mitochondrial function contributes to excessive ROS production, chronic inflammation, and hepatocyte apoptosis, key mechanisms that drive disease progression.^2^ By enhancing mitochondrial function, reducing oxidative stress, and inhibiting apoptosis, SHLP2 and SHLP3 may counter these processes, thereby protecting hepatic tissue and alleviating the cellular damage and metabolic dysregulation associated with MAFLD. In particular, SHLP2 has been shown to improve insulin sensitivity and regulate energy homeostasis through both peripheral and central mechanisms. In HFD–fed mice, systemic administration of SHLP2 via intraperitoneal (IP) injection improved glucose tolerance and insulin signaling, reduced hepatic lipogenic gene expression and steatosis, increased energy expenditure, and suppressed food intake.^65^ Central administration of SHLP2 via intracerebroventricular (ICV) injection directly activated hypothalamic pro-opiomelanocortin (POMC) neurons, resulting in appetite suppression and increased thermogenesis.^65^ Notably, peripherally administered SHLP2 (IP) was also detectable in the cerebrospinal fluid and activated the same hypothalamic POMC neurons. These effects are mediated by SHLP2 binding to the chemokine receptor 7 (CXCR7), which triggers the mitogen-activated protein kinase (MAPK)/ERK1/2 signaling cascade in POMC neurons, integrating central and peripheral regulation of energy balance^65^ (Figure 2). Collectively, these findings suggest that the peripheral metabolic benefits of SHLP2, including improved glucose tolerance and reduced hepatic lipid accumulation, are partly coordinated through central activation of the hypothalamic POMC/CXCR7 pathway.^65^ Moreover, the metabolic effects of SHLP2 extend to hepatic glucose regulation. In hyperinsulinemic-euglycemic clamp studies, ICV infusion of SHLP2 in rats significantly suppressed hepatic glucose production, as indicated by an increased glucose infusion rate required to maintain euglycemia^11^ (Figure 4B). Together, these results highlight a broader role for SHLP2 in systemic metabolic regulation and suggest potential therapeutic relevance for metabolic disorders such as MAFLD.

Among the SHLP family, SHLP2 is the most well characterized, while SHLP3 has been studied to a lesser extent. In contrast, the biological functions of SHLP1, SHLP4, and SHLP6 remain largely unknown. Expression profiling indicates that these peptides are highly expressed in the liver,^11^ suggesting that they may have physiological relevance in hepatic metabolism. However, direct experimental evidence delineating their roles in liver function or disease is currently lacking. Given the emerging importance of MDPs in regulating metabolic homeostasis and cellular stress responses, it is imperative to further investigate the functional roles of the lesser-known SHLPs. Such studies could provide novel insights into mitochondrial signaling networks and identify new therapeutic targets for MAFLD and related liver disorders.

## MOTS-c: MDP LINKING ENERGY METABOLISM TO LIVER HEALTH

MOTS-c is a 16-amino-acid peptide encoded by the mitochondrial MT-RNR1 gene^10^ (Figure 1). Unlike most mitochondrial proteins, which are transcribed and translated within the mitochondrial matrix, MOTS-c is uniquely translated in the cytoplasm.^10^ This is due to the presence of a mitochondria-specific stop codon (AGG) immediately following the start codon, which would prematurely terminate translation if processed by mitochondrial ribosomes.^10^^,^^66^ Instead, the transcript is exported to the cytoplasm, where it is translated using the standard genetic code^10^ (Figure 2). Under conditions of cellular stress, such as oxidative damage or nutrient deprivation, MOTS-c can translocate to the nucleus, where it modulates gene expression and promotes adaptive stress responses.^10^ In contrast to Humanin, which has NUMTs, no NUMT-derived variants of MOTS-c have been identified.^10^ This suggests that its expression is entirely dependent on the integrity and transcriptional activity of the mitochondrial genome, underscoring its utility as a sentinel of mitochondrial health.^10^ Circulating MOTS-c has been detected in plasma and exerts endocrine-like effects on systemic metabolism, including enhanced insulin sensitivity and glucose utilization.^10^ Despite these systemic actions, a specific cell-surface receptor for MOTS-c has not yet been identified, and direct evidence for autocrine or paracrine signaling remains limited. In HEK293 cells exposed to fluorescein isothiocyanate (FITC)-conjugated MOTS-c, the peptide rapidly localized to both mitochondria and nuclei within 30 minutes, displaying a punctate distribution within the nucleus.^8^ These findings suggest that the systemic effects of circulating MOTS-c are likely mediated through cellular uptake rather than classical receptor-dependent signaling mechanisms (Figure 2).

At the molecular level, MOTS-c regulates cellular energy homeostasis by modulating AMPK, a key regulator of metabolic stress responses.^10^ Specifically, MOTS-c inhibits enzymes involved in one-carbon metabolism, including methylenetetrahydrofolate dehydrogenase 2 (MTHFD2) and serine hydroxymethyltransferase 2 (SHMT2). This inhibition disrupts the folate cycle, leading to depletion of 5-methyl-tetrahydrofolate (5-MTHF), an active form of folate. Reduced 5-MTHF impairs de novo purine biosynthesis (DNPB), causing intracellular accumulation of 5-aminoimidazole-4-carboxamide ribonucleotide (AICAR), an endogenous AMPK activator.^10^ Intracellularly, AICAR is converted to AICAR monophosphate (ZMP), an AMP analog that binds the γ-subunit of AMPK, promoting its allosteric activation and phosphorylation by the upstream kinase liver kinase B1 (LKB1).^67^ Through this mechanism, MOTS-c–induced AICAR accumulation enhances AMPK activity, effectively mimicking a cellular energy-deficient state even under normal ATP conditions^10^ (Figure 3C).

Interestingly, AMPK activation also facilitates the nuclear translocation of MOTS-c, where it interacts with transcription factors such as activating transcription factor 1 (ATF1) and nuclear factor erythroid 2–related factor 2 (NRF2) to regulate antioxidant response element (ARE)-driven genes, thereby enhancing the cellular antioxidant defense system.^8^^,^^38^ Notably, overexpression of MOTS-c in HEK293 cells leads to a significant reduction in basal mitochondrial oxygen consumption rate (OCR), suggesting an adaptive downregulation of mitochondrial respiratory activity.^10^ This modulation likely serves to optimize energy efficiency and limit ROS production, thereby minimizing oxidative damage. Intriguingly, studies using MOTS-c mutants deficient in nuclear translocation, as well as nuclear loss-of-function variants, demonstrated that the suppression of OCR persisted despite impaired nuclear localization.^8^ These findings strongly indicate that MOTS-c primarily regulates mitochondrial respiration through direct interactions with mitochondria, rather than via nuclear transcriptional control. The translocation of MOTS-c to the nucleus in response to metabolic or oxidative stress, where it acts as a retrograde signaling molecule conveying mitochondrial status, highlights its role as a crucial mediator linking mitochondrial activity to nuclear gene expression, thereby coordinating adaptive responses that maintain mitochondrial function and overall cellular homeostasis (Figure 3C).

Furthermore, a recent investigation elucidated a pivotal role for MOTS-c in the pathogenesis and progression of MAFLD and its more severe form, MASH, primarily through modulation of the mitochondrial apoptotic machinery involving BAX and BCL2 family proteins.^14^ In murine models of MASH, both chronic preventive and acute therapeutic administration of MOTS-c markedly attenuated hepatic steatosis, decreased hepatocyte apoptosis, and ameliorated inflammation and fibrosis.^14^ Mechanistic studies have revealed that MOTS-c exerts these hepatoprotective effects by directly binding to the BH3 domain of the anti-apoptotic protein BCL2, thereby stabilizing BCL2 by inhibiting its ubiquitination and subsequent proteasomal degradation.^14^ This stabilization potentiates the anti-apoptotic activity of BCL2, effectively preventing activation of the pro-apoptotic effector BAX, which reduces hepatocyte apoptosis and limits the progression of liver injury in MASH.^14^ The critical involvement of BCL2 in MOTS-c-mediated hepatoprotection was further confirmed by experiments demonstrating that adeno-associated virus (AAV)-mediated knockdown or pharmacological inhibition of BCL2 abolished the beneficial effects of MOTS-c in the MASH model^14^ (Figure 3C).

Beyond apoptosis regulation, MOTS-c also modulates fibrogenic signaling pathways in the liver. In rat models of type 2 diabetes-associated liver fibrosis, MOTS-c treatment suppressed activation of the transforming growth factor-beta 1 (TGF-β1)/mothers against decapentaplegic homolog 2, 3, and 4 (Smad2/3/4) signaling cascade, a central pathway driving hepatic stellate cell (HSC) activation and extracellular matrix deposition.^13^ This suppression resulted in decreased collagen production and improved overall liver function, highlighting the anti-fibrotic potential of MOTS-c. Complementary in vitro studies using hepatic stellate cells (LX-2), normal hepatocytes (LO-2), and hepatocellular carcinoma cells (HepG2) demonstrated that MOTS-c overexpression or exogenous peptide treatment effectively reduced intracellular ROS levels, upregulated expression of antioxidant genes, and downregulated pro-fibrotic gene expression.^13^ These findings collectively suggest that MOTS-c mitigates oxidative stress and fibrogenesis, both of which are key drivers of liver disease progression. Furthermore, MOTS-c has been shown to suppress the production of pro-inflammatory cytokines, thereby dampening hepatic inflammation, a critical factor in the transition from simple steatosis to MASH.^14^ The cumulative evidence indicates that MOTS-c exerts multifaceted hepatoprotective effects by modulating apoptosis, oxidative stress, fibrosis, and inflammation (Figure 4C). These diverse mechanisms underscore MOTS-c as a highly promising therapeutic candidate for metabolic liver diseases such as MAFLD and MASH. Continued mechanistic elucidation and translational research into MOTS-c may ultimately facilitate the development of novel mitochondria-targeted therapies designed to prevent and treat these increasingly prevalent hepatic disorders, including ALD.

A specific mtDNA polymorphism, m.1382A>C, results in a lysine-to-glutamine substitution at position 14 (K14Q) in the MOTS-c peptide.^68^ This variant produces a partially bioinactive form of MOTS-c with diminished capacity to enhance insulin sensitivity and reduce fat mass.^68^ It has been linked to increased visceral adiposity and an elevated risk of type 2 diabetes, particularly in East Asian men with sedentary lifestyles.^68^ This observation adds complexity to the role of MOTS-c, given that mtDNA mutations are commonly associated with aging and metabolic disorders.^69^^–^^72^ Consequently, relying solely on serum MOTS-c levels as a biomarker may be misleading, as such measurements do not distinguish between the wild-type and mutant forms. Future studies should therefore incorporate approaches capable of detecting MOTS-c variants to more accurately assess their clinical significance.

## EMERGING DIRECTIONS AND CRITICAL GAPS

MDPs have emerged as a distinct class of endogenous bioactive molecules with significant implications for the pathogenesis and treatment of liver diseases. These peptides, including Humanin, MOTS-c, and SHLPs, exert multifaceted effects on key cellular processes that sustain hepatic homeostasis, such as regulation of metabolic balance, attenuation of oxidative stress, and modulation of pro-inflammatory signaling pathways (Table 1). While substantial progress has been made in elucidating the intracellular functions of MDPs, their extracellular mechanisms of action remain largely unknown. In particular, the receptor-mediated signaling pathways engaged by MDPs in hepatocytes and non-parenchymal liver cells are poorly characterized, especially within the complex microenvironment of chronic liver disease. A comprehensive understanding of how MDP expression across different hepatic cell types, together with circulating MDP levels, coordinately influences cellular responses is therefore critical for delineating their contribution to liver disease pathophysiology. Furthermore, distinguishing the effects of intracellularly expressed MDPs in maintaining cellular homeostasis from those mediated by circulating or exogenously administered MDPs will provide important insight into their internal and external mechanisms of action.

**TABLE 1:** Comparative overview of MDPs: origin, cellular targets, and biological function

Peptide	Year discovered	Length (aa)	mtDNA locus	Intracellular targets and effects	Extracellular receptors	Effect on the liver
Humanin	2001^9^	21/24	*MT-RNR2* (16S rRNA gene)	BAX; inhibits apoptosis; activates chaperone-mediated autophagy (CMA)	FPRL1; CNTFR-α/gp130/WSX-1 Tripartite Complex	Mitigates hepatic steatosis, enhances insulin sensitivity, and is hepatoprotective
SHLP1	2016^11^	24	*MT-RNR2* (16S rRNA gene)	Targets and effects are not well defined	Not well defined	Detected in the liver; physiological role unknown
SHLP2	2016^11^	26	*MT-RNR2* (16S rRNA gene)	Mitochondrial Complex I; inhibits apoptosis; reduces ROS; enhances mitochondrial function	CXCR7	Enhances insulin sensitivity; suppresses HFD-induced hepatic lipogenic gene expression and steatosis
SHLP3	2016^11^	38	*MT-RNR2* (16S rRNA gene)	Inhibits apoptosis; reduces ROS; enhances mitochondrial function	Not well defined	Role not clearly defined
SHLP4	2016^11^	26	*MT-RNR2* (16S rRNA gene)	Targets not well defined; promotes cell proliferation	Not well defined	Detected in the liver; physiological role unknown
SHLP5	2016^11^	24	*MT-RNR2* (16S rRNA gene)	Targets and effects are not well defined	Not well defined	Not detected in liver; potential inducibility and role remain unknown
SHLP6	2016^11^	20	*MT-RNR2* (16S rRNA gene)	May promote apoptosis	Not well defined	Detected in the liver; physiological role unknown
MOTS-c	2015^10^	16	*MT-RNR1* (12S rRNA gene)	AMPK activation, BCL2 stabilization, regulates ARE gene expression; inhibits folate metabolism/purine biosynthesis	Not well defined; primarily considered to act intracellularly	Reduces steatosis, apoptosis, and inflammation in MASH; exhibits anti-fibrotic effects

Abbreviations: aa, amino acid; AMPK, AMP-activated protein kinase; ARE, antioxidant response element; BAX, BCL2-associated X protein; BCL2, B-cell lymphoma 2; CNTFRα, ciliary neurotrophic factor receptor alpha; CXCR7, chemokine receptor 7; FPRL1, formyl peptide receptor-like 1; gp130, glycoprotein 130; HFD, high-fat diet; MASH, metabolic dysfunction–associated steatohepatitis; MDPs, mitochondria-derived peptides; MOTS-c, Mitochondrial Open-reading-frame of the Twelve S rRNA-c; MT-RNR1, mitochondrial rRNA 1; MT-RNR2, mitochondrial rRNA 2; rRNAs, ribosomal RNAs; ROS, reactive oxygen species; SHLPs, small Humanin-like peptides.

Reduced MDP expression has been associated with pathological conditions such as aging, obesity, and diabetes,^5^^,^^11^^,^^57^^,^^73^^,^^74^ potentially reflecting mtDNA depletion commonly observed in these states.^56^^,^^75^^–^^77^ Interestingly, circulating levels of MOTS-c and SHLP2 are elevated in non-diabetic individuals with increased android and hepatic fat accumulation.^16^ Similarly, in mice, plasma levels of these MDPs rise in response to a high-fat, choline-deficient diet-induced hepatic steatosis.^16^ These findings suggest that MDP upregulation represents an adaptive, hormetic response to metabolic stress, functioning to restore cellular and systemic metabolic balance. This increase may also reflect a compensatory mechanism driven by enhanced mitochondrial biogenesis and increased mtDNA copy number during early stages of liver injury.^78^^,^^79^ However, the regulatory mechanisms controlling MDP transcription and translation remain poorly understood. In particular, for MOTS-c and Humanin (the 24–amino acid form), which rely on cytosolic translation of mitochondrially encoded transcripts, the processes mediating mRNA export from mitochondria to the cytoplasm remain incompletely defined. Moreover, the mechanisms underlying MDP secretion are largely unknown, highlighting a fundamental gap in our understanding of how MDP expression and secretion are regulated. Elucidating these pathways, along with identifying the primary tissue sources contributing to circulating MDP pools, is essential for understanding their physiological and pathological roles in liver and systemic metabolism.

Damage to mtDNA, through mutations or depletion, can compromise the assembly and function of ETC complexes, impair ATP synthesis, and increase ROS generation.^4^^,^^80^ These mitochondrial dysfunctions play a central role in metabolic and inflammatory diseases, including MASLD and ALD.^3^^,^^81^ Furthermore, mtDNA damage may directly impair MDP expression and function.^5^ Mutations or deletions in mtDNA can disrupt MDP synthesis or produce dysfunctional peptides, thereby compromising critical regulatory pathways of mitochondrial function.^5^^,^^68^ Notably, the mtDNA polymorphism m.1382A>C, found in East Asian men with type 2 diabetes, induces a K14Q substitution in MOTS-c, generating a partially bioinactive peptide, whereas the Parkinson disease-protective polymorphism m.2158T>C induces a K4R substitution in SHLP2, producing a more stable and protective peptide.^68^^,^^82^ Given the high susceptibility of mtDNA to oxidative damage,^83^ mutations in MDP-coding regions may contribute to disease pathogenesis. This highlights the need to investigate whether similar mtDNA variants occur in other MDPs, particularly in chronic liver diseases characterized by elevated mitochondrial ROS production and mtDNA instability.^2^

## CONCLUSIONS

MDPs, with their diverse and potent biological actions, represent a novel and mechanistically grounded therapeutic avenue for liver disease. Targeting MDP signaling pathways offers the potential to transform current treatment paradigms, shifting from symptomatic management to precision strategies that directly address the mitochondrial and metabolic dysfunctions underlying disease progression. MDP-based therapies may provide unique advantages, including low toxicity and the potential for early intervention in disease pathogenesis. Despite their promise, significant gaps remain in our understanding of how MDPs influence liver pathology at the molecular and cellular levels. Key questions include defining receptor-mediated signaling networks, elucidating crosstalk among mitochondrial, nuclear, and cytosolic pathways, and determining the mechanisms governing peptide trafficking and distribution in vivo. Addressing these challenges will be critical for translating MDP biology into clinically viable therapies. By harnessing their intrinsic signaling properties, MDPs have the potential to serve as targeted, durable interventions that directly counteract the molecular drivers of MAFLD, ALD, and related hepatic disorders.
